# Tumor necrosis factor alpha and palmitate simulate bovine fatty liver disease in vitro when using abattoir-derived primary bovine hepatocytes isolated by a novel nonperfusion method

**DOI:** 10.3168/jdsc.2022-0263

**Published:** 2022-09-03

**Authors:** E.D. Testroet, S. Choudhary, R.K. Choudhary, D.C. Beitz, M. Du

**Affiliations:** 1 Department of Animal and Veterinary Sciences, University of Vermont, Burlington 05446; 2 Department of Animal Science, Iowa State University, Ames 50011; 3 Department of Animal Sciences, Washington State University, Pullman 99163

## Abstract

Hepatic lipidosis (i.e., fatty liver) is a common periparturient disease in high-producing dairy cattle affecting nearly 50% of cows to some degree and costing an estimated 60 million dollars annually. Large animal studies are costly, labor intensive, and are not well suited to mechanistic studies. Traditionally, mechanistic studies employ in vitro methodologies, utilizing established cell lines or primary cell culture methods. However, with dairy cattle, established hepatic cell lines do not exist, and methods for primary cell culture studies typically involve complicated procedures that often utilize very young animals (typically bull calves). Several previously published papers have used abattoir-derived tissues as a source of primary cells; however, a simple method utilizing simple culture media has yet to be presented. In addition, we sought to develop a way to replicate the syndrome of fatty liver disease “in a dish” using adult cattle that should more closely represent the physiology of the periparturient dairy cow. Herein we present a non-perfusion-based method that results in robust growth and proliferation of abattoir-derived bovine hepatocytes that demonstrate lipid loading, elevated lactate dehydrogenase leakage, and cytotoxicity as demonstrated by elevated caspase 3/7 expression consistent with in vivo physiology of the periparturient dairy cow with fatty liver disease.

## Highlights

• A method for culturing hepatic cells from abattoir-derived dairy cow liver is presented.
• This method utilizes adult dairy cow tissue that is readily available.
• Because the culture comprises all hepatic cell types in comparable proportions to those in the live dairy cow, we expect this method to be more representative of in vivo fatty liver disease.
• Our method is simple, novel, consistent with other cell models, and consistent with fatty liver disease in dairy cows during the periparturient period.
• We present a novel method to pursue mechanistic studies of fatty liver in the periparturient dairy cow.

## Graphical Abstract

**Figure:**
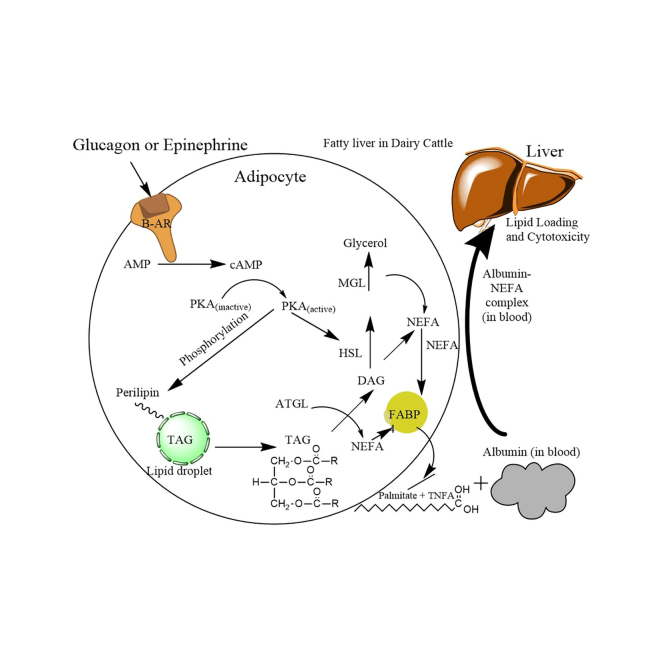
**Summary:** A schematic representation of lipid mobilization in the transition dairy cow is shown. Dysregulation or hypermobilization of nonesterified fatty acids (NEFA) during the periparturient period result in increased blood NEFA and tumor-necrosis factor alpha (TNFA) concentrations in blood. Increased concentrations of NEFA and TNFA overwhelm the capacity of the liver to dispose of lipids in the periparturient dairy cow, resulting in lipid loading and cytotoxicity and, ultimately, fatty liver disease.

Hepatic lipidosis (i.e., fatty liver) is a prevalent disease in the high-producing transition dairy cow, particularly when the cow is over-conditioned, affecting nearly half of all dairy cows to some degree (Bobe et al., 2004). When high-producing dairy cattle are over-conditioned, the mobilization of triglycerides from the adipose tissue overwhelms the capacity of the liver to both oxidize and mobilize those fatty acids. When the capacity of the liver to utilize fatty acids, either through export or oxidation, is overwhelmed, accumulation of triglycerides in the liver occurs, which perturbs many metabolic functions. Fatty liver exacerbates other periparturient diseases of the transition dairy cow (e.g., ketosis, metritis, mastitis) and results in losses to the producer (>$60 million annually; Bobe et al., 2004), as well as impaired animal welfare and decreased sustainability. Numerous studies have focused on the mechanisms of development of hepatic lipidosis in various animal models; however, the physiology of the ruminant animal compared with the rodent and human are quite different. In the ruminant, de novo fatty acid synthesis occurs primarily in the adipose tissue, utilizing short-chain fatty acids as a substrate. In the human and rodent, hepatic de novo lipid synthesis occurs primarily in the liver or in the liver and the adipose tissue, respectively. In addition, the substrate for production of lipid in the liver of human and rodent is primarily carbohydrate, in contrast with that in the ruminant (Laliotis et al., 2010).

Much research has been done on the pathology and etiology of fatty liver in the periparturient dairy cow, but the disease is still prevalent, indicating that further understanding of the mechanisms of disease development and progression are necessary (Bobe et al., 2004).

A few in vitro culture methods for bovine hepatic cells have been presented, but they are complicated in several ways because they involve one or more of the following: collagenase perfusion (Berry and Friend, 1969; Witte et al., 2019) or complex culture media (Ehrhardt and Schmicke, 2016) often supplemented with exogenous growth factors or steroids (Giantin et al., 2012). A second consideration is that most previous research used male calves (Donkin and Armentano, 1993) because of availability and cost, or female newborn calves (Li et al., 2018) that are more costly and may not represent the physiology of the adult cow, and additionally will have to be euthanized. Moreover, abattoir-derived liver has been utilized for primary cell culture in several species such as equine (Stefanski et al., 2013), porcine (Gerlach et al., 1993), and bovine species (Panda et al., 2015; Ehrhardt and Schmicke, 2016). Because of the lack of available established cell lines or established *simple* primary cell culture methodologies and with the need for further mechanistic studies in mind, we sought to develop an in vitro model of bovine hepatic lipidosis. Our objective was to develop a simple model for the collection, culture, and experimentation with primary Holstein heifer liver cells collected from the left lobe of the liver in the abattoir. We hypothesized that through optimization of culture conditions and cell isolation we could reliably and easily culture liver cells from culled Holstein dairy cattle and recreate the phenotype of hepatic lipidosis that could be used for mechanistic studies to support live animal observations. The abattoir was chosen for ease and availability of tissue collection, although the applicability of this culture protocol should be suitable for tissue collected from live animals via puncture biopsy.

Once a suitable culture methodology was established, a method for inducing lipidosis in vitro was established and is described below. In addition to the convenience and simplicity of this culture method and because this method is a culture of all liver cells rather than a purified fraction, this model should more accurately represent in vivo conditions. Finally, by utilizing cells derived from culled female Holstein cattle, the physiological response should be more similar than using neonates as a source of cells and the number of live animal experiments required for doing mechanistic studies could be decreased.

This article does not contain any studies with human or animal subjects and did not require IACUC or IRB approval. Liver (∼10 g) was collected immediately after slaughter from culled Holstein heifers (approximately 635 kg and 22–23 mo old). These heifers were healthy and were not culled because of any disorder. Tissue was transferred from the abattoir (within 1 h postslaughter) to the laboratory on ice and processed immediately with the following protocol. 1. Immediately after collection, put liver tissue sample in sterile PBS with 1% penicillin and streptomycin (Sigma Aldrich) on ice until cell isolation. 2. In disposable sterile Petri dishes, rinse the liver tissue in PBS 3 times. 3. Transfer the entire liver sample to a disposable sterile Petri dish containing digestion medium. Digestion medium: 200 U/mL Collagenase Type II, Roche #110888741043 in sterile serum-free low-glucose Dulbecco Modified Eagle Medium‎ (**DMEM**) supplemented with 3.7 g/L sodium bicarbonate (Gibco, Thermo Fisher Scientific). The optimal tissue to digestion medium ratio should be determined by the user, but 20 mL of digestion medium is typically enough for 10 g of tissue. 4. Mince liver tissue in digestion medium with sterile micro scissors. 5. Digest tissue at 37°C for 30 min with agitation in a sealed 50-mL conical vial in a shaking incubator (90 rpm; New Brunswick Scientific Excella E24 Incubator Shaker). Shaking is important to keep cells oxygenated and facilitates digestion. In addition, sealing the vial with parafilm (Bemis NA) will help prevent spillage or contamination. 6. After the digestion for 30 min, pass digested tissue through a 1-mL pipette tip several times to facilitate digestion (cutting a bit off the pipette tip will prevent clogging). 7. Stop digestion by adding a volume of culture medium equal to the volume of digestion medium. Culture medium: DMEM with 10% fetal bovine serum (**FBS**; Atlas Biologicals), 3.7 g/L supplemental sodium bicarbonate, and 1% penicillin and streptomycin. 8. Allow the tissue fragments to settle (∼1 min) and remove the supernatant to a new 50-mL conical vial. Wash the undigested tissue fragments in with 10 mL of FBS and DMEM and transfer this supernatant to the same new conical vial. Discard the undigested tissue. 9. Centrifuge at 500 × *g* for 5 min at 4°C. 10. Remove supernatant and resuspend cell pellet by using gentle pipetting in 5 mL of serum-free low-glucose DMEM with 3.7 g/L supplemental sodium bicarbonate and 1% penicillin and streptomycin. 11. Centrifuge at 500 × *g* for 5 min at 4°C. 12. Repeat step 10 and 11 twice. 13. Remove supernatant and resuspend tissue pellet in culture medium. 14. Seed freshly isolated hepatic cells in 100 mm of gelatin-coated cell culture plates (Thermo Fisher Scientific) in culture media and incubate 48 h at 37°C, 5% CO_2_, 95% O_2_. 15. Change media after 48 h and wash with PBS. 16. Change media after 4 d to allow fibroblast growth factors to accumulate. After the 4-d incubation, fresh culture medium should be applied every 48 h. 17. Allow cells to grow until near 80 to 90% confluence, wash plate with PBS, and digest cells with trypsin and EDTA (Sigma Aldrich) for 3 min at 37°C. 18. Stop digestion by adding a volume of culture medium equal to the amount of trypsin and EDTA used for digestion. 19. Using mechanical disruption, transfer cells to a new conical tube, centrifuge at 500 × *g* for 5 min at 4°C, remove supernatant, and resuspend in culture medium. 20. Seed hepatic cells (approximately 2.4 × 10^5^ cells/mL) into gelatin from porcine skin (Sigma Aldrich) coated experimental cell culture plates (Greiner Bio-One). 21. Cells should be ready for treatment after 24 h when fully confluent.

Initially, Williams' medium E was used for protocol development because it was developed for the culture of rat primary hepatocytes (Williams et al., 1971). However, as mentioned earlier, the physiological function of bovine liver compared with that of rodent liver is different, and, upon comparison between DMEM and William's E basal medium, it was determined that DMEM supported better growth of viable hepatic cells (results not shown). Therefore, hepatic cell culture medium utilized for growth and maintenance is DMEM supplemented with 3.7 g/L sodium bicarbonate, 10% FBS, and 1% penicillin and streptomycin.

Tumor necrosis factor α (**TNFA**, 100 µg, Acro Biosystems) was reconstituted in 500 µL of sterile water per manufacturer instructions to form a 200 µg/mL stock solution, aliquoted, and stored at −70°C until use with the final concentration being 20 ng/mL. This concentration was chosen based upon previously published cell culture with bovine mammary epithelial cells (Leibowitz and Cohick, 2009).

Because palmitate is the most prevalent fatty acid in the liver of overfed dairy cows 0.5 wk after calving (Rukkwamsuk et al., 2000), a 300 m*M* sodium palmitate stock solution was prepared by saponifying palmitic acid (Sigma Aldrich) in equimolar NaOH at 65°C. Saponification was necessary because palmitic acid is inherently very insoluble in aqueous solutions. This stock solution was then further diluted with ultrapure water to 30 m*M*, which was heated at 80°C for at least 30 min and added directly to 10% FBS and DMEM with the final concentration being 0.4 m*M*. The palmitate then was allowed to complex with the albumin present in the FBS component of the culture medium for at least 60 min at room temperature before treatment. The palmitate concentration was chosen to elicit maximal intracellular lipid loading without inducing massive cytotoxicity (Wobser et al., 2009).

Cellular morphology was observed by transillumination imaging. Since cultured hepatocytes tend to undergo dedifferentiation over time in culture (Elaut et al., 2006), losing hepatic cell specific markers and viability, viability of hepatic cells was assessed by using a commercial kit (ReadyProbes Cell Viability Imaging Kit, Blue/Green, Invitrogen) and trypan blue staining. To assess cytotoxicity, quantitative measurement of lactate dehydrogenase (**LDH**) leakage from damaged cells was conducted (Pierce LDH cytotoxicity kit, Thermo Fisher Scientific). Cells were cultured for 2 to 3 d and then treatment was initiated (palmitate, TNFA, and palmitate plus TNFA). Each treatment was replicated 4 times (technical replicates) in a 96-well plate, and the experiment was repeated twice. After 24 h of treatment culture, the medium was collected for LDH quantification. Addition of sterile water and 10× lysis buffer to cells represented baseline LDH and maximum LDH leakage, respectively. After addition of reaction mixture to the collected culture media (50 µL) in a 96-well plate, stop buffer was added and absorbance was measured per the manufacturer's instructions. Absorbance was measured at 490 and 680 nm, the difference was calculated, and the mean value was used for quantification of LDH.

Staining of bovine primary liver cells were done as described elsewhere (Choudhary and Capuco, 2012) without the antigen retrieval step. Hepatocytes and stellate cells were identified using E-cadherin and α-smooth muscle primary antibodies, respectively. About 10 randomly selected photomicrographs of each group were captured at 100× magnification using the EVOS M5000 Imaging System and marker positive cells were quantified using ImageJ. Percentage of positive cells were quantified out of the total 4′,6-diamidino-2-phenylindole (**DAPI**)-positive cells nuclei.

The primary bovine hepatic cell cultures used for apoptosis assay were done in duplicate using 24-well culture plates. After 2 to 3 d in culture, cells of all treatment groups were serum deprived overnight followed by incubation with palmitate, TNFA, and palmitate plus TNFA. The control wells received fresh medium with no treatment. After 24 h of incubation, the cells were labeled with fluorescent inhibitor of caspases (**FLICA**; Image-iT LIVE Green Caspase-3 and -7 Detection Kit; Thermo Fisher Scientific). The FLICA covalently binds with caspase-3 and -7 amino acid sequences and emits a green fluorescent signal, indicating apoptotic cells. After washing with PBS, 2 drops of aqueous mounting media with DAPI (Vectamount, Vector Lab) was applied onto each well. Cells were viewed immediately and analyzed under the EVOS M5000 Imaging System (Thermo Fisher Scientific). For each experimental condition, at least 20 random microscopic fields in blue and green channels were captured in duplicate cultures. The number of cells stained with FLICA and DAPI was determined from each image using ImageJ (National Institutes of Health). The percentage of green apoptotic cells in relation to the total DAPI-positive cells was calculated.

Hepatic cells collected from 3 Holstein dairy heifers were grown until confluence (∼6 d) in 100-mm gelatin-coated cell culture dishes (Thermo Fisher Scientific) and then subcultured into 24-well culture plates. After 2 to 4 d, cells were cultured in presence of (1) 0.4 m*M* palmitate, (2) 20 ng/mL TNFA, or (3) 0.4 m*M* palmitate plus 20 ng/mL TNFA in culture medium. Cells grown in culture medium alone served as the control. Cells were serum deprived overnight before applying any treatments. After 24 h of treatment, relative lipid accumulation was determined by Oil Red O stain (EMD Millipore). Staining results were quantified as the number of Oil Red O stain-positive cells over the total cells counted in the photomicrographs. In each treatment and control groups, 20 images from the 4 wells were captured using EVOS M5000 Imaging System (Thermo Fisher Scientific). The total number of nuclei was counted using ImageJ (National Institutes of Health) and cells showing red lipid stains were counted manually. Finally, the percentage of lipid-stained cells was calculated.

Data were analyzed using the MIXED procedure of SAS version 9.4 (SAS Institute Inc.) with the model including individual animal and treatment as fixed effects and animal within treatment as a random effect. Means were separated by using an *F*-protected LSMEANS test. The percentage positive cells (lipid staining and apoptosis) were transformed to the square root of proportions and arsin transformed, then analyzed using a one-way ANOVA. Data were considered significant if *P* < 0.05.

As can be seen in Figure 1, 24-h treatment with palmitate with or without the presence of TNFA induced an approximately 3- to 5-fold increase of LDH leakage; however, TNFA alone was not significantly different from control, indicating that 0.4 m*M* palmitate alone was enough to induce significant increases in cytotoxicity consistent with known effects of palmitate on cell death (Xu et al., 2015). Because the model is a mixed cell model, immuno-identification of cell types was undertaken (Figure 2), confirming that the ratio of hepatocytes to stellate cells (nonparenchymal cells) approximates that of the liver. Tumor necrosis factor α (**TNFA**) has been shown to be strongly associated with fatty liver in dairy cows in vivo (Ametaj et al., 2005) and palmitate is the primary fatty acid found in circulation of dairy cows with fatty liver (Rukkwamsuk et al., 2000). Therefore, it appears that the physiology of the dairy cow with fatty liver is consistent with the so-called “2-hit hypothesis” where TNFA is elevated and circulating NEFA are increased (Day and James, 1998). Therefore, a combination of palmitate and TNFA was also included as a treatment. As expected, based on the in vivo phenotype of dairy cattle with fatty liver, culturing cells with palmitate and TNFA induced an approximate 4-fold increase in expression of the apoptosis markers caspase 3/7 (Figure 2) consistent with limited in vivo research in periparturient dairy cattle with hepatic lipid accumulation and ketosis (Du et al., 2017). Furthermore, while palmitate treatment increased lipid accumulation roughly 4-fold above control, TNFA exacerbated lipid accumulation increases to 10 to 15 times that of control (Figure 3).

**Figure 1:**
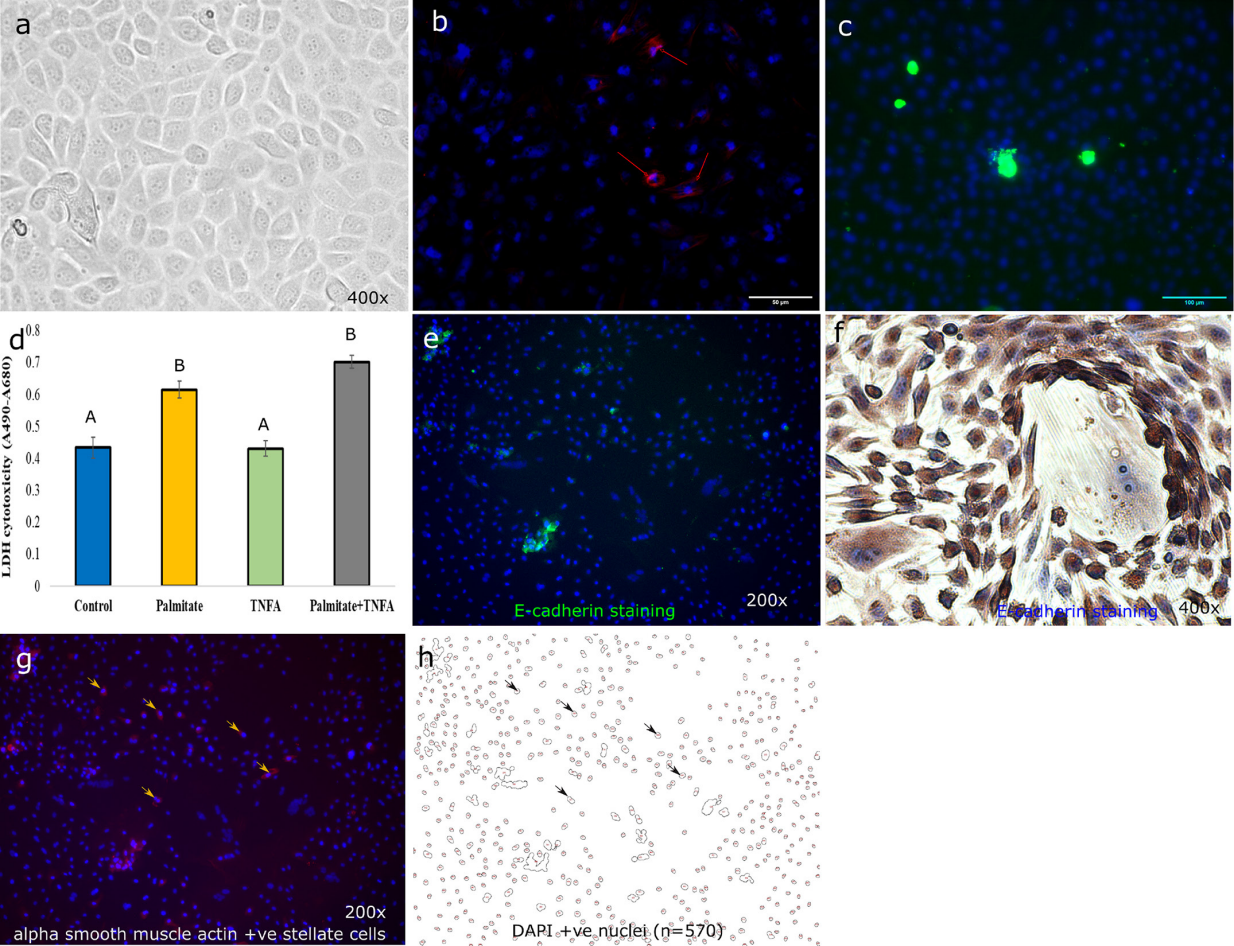
Cellular and immunohistochemical phenotype of a confluent culture of mixed bovine liver and cytotoxicity assay. A representative image showing a transillumination image of cuboidal-shaped hepatocytes (a). Stellate cells in culture stained positive with α smooth muscle acting (red arrows, b) and more than 95% of the cells were alive in the culture (c). Estimation of cytotoxicity was evaluated using lactate dehydrogenase (LDH) activity (difference in absorbance of 490 and 680 nm; d). Each group, except the control group, was incubated in medium supplemented with 0.4 m*M* palmitate or tissue necrosis factor α (TNFA) or 0.4 m*M* palmitate plus 20 ng/mL TNFA for 24 h. Data are shown as the means ± SEM (n = 8). Expression of E-cadherin as a marker of hepatocytes was not evident in immunocytochemical staining (e) but evident under the bright field (f). Alpha-smooth muscle actin-marked stellate cells (red arrows, g) were present in approximately 5% of the total cells. Counter-staining of cells was done using 4′,6-diamidino-2-phenylindole (blue nuclei) in fluorescent staining, and the total number of nucleic was counted by ImageJ (National Institutes of Health) using the cell counter plugin (h). Columns with different letters (A, B) differ significantly (*P* < 0.05). +ve = positive.

**Figure 2:**
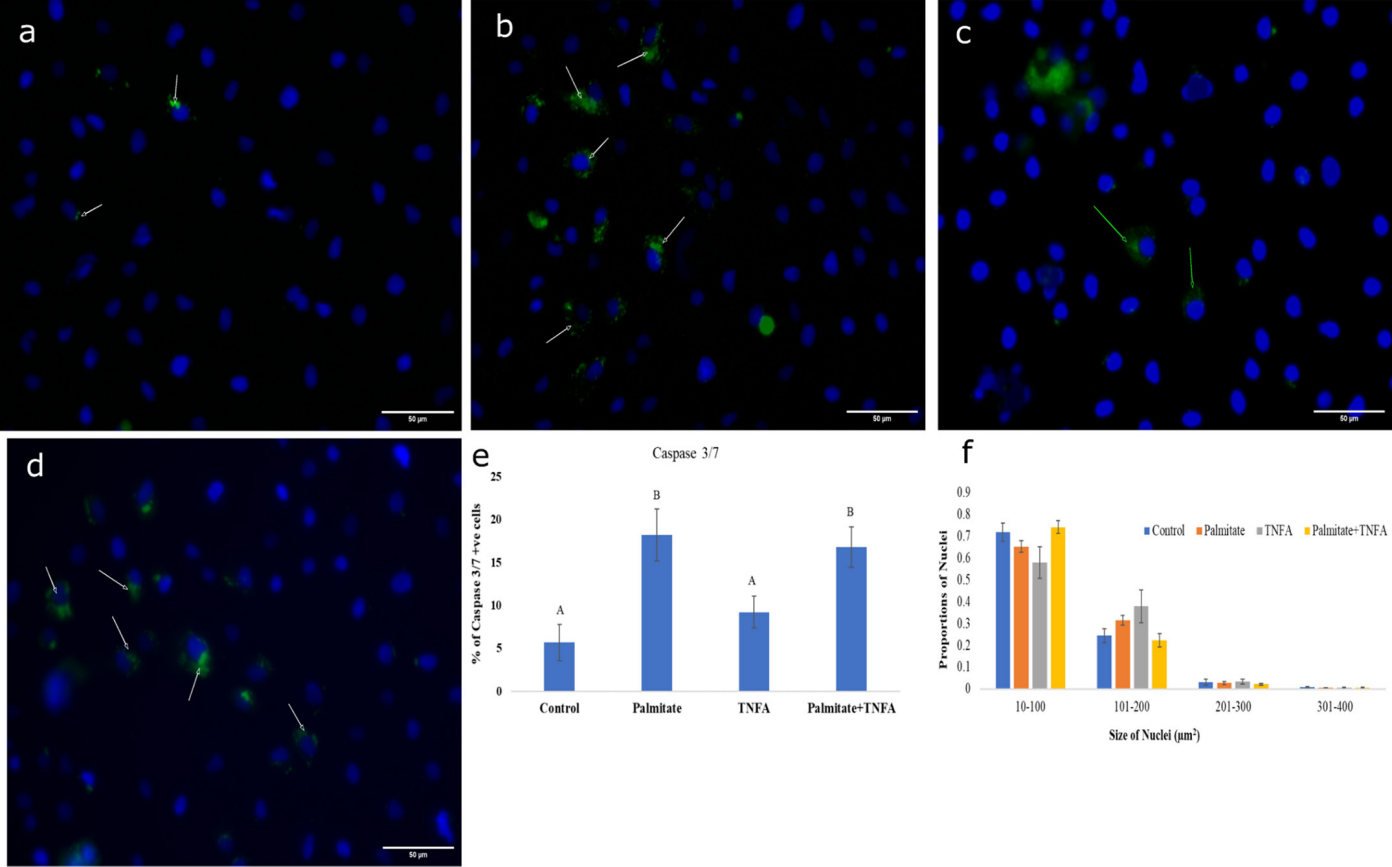
Caspase 3/7 activity in bovine primary liver cells after 24 h of treatment. (a) Primary bovine hepatocytes were incubated in 10% fetal bovine serum/Dulbecco's Modified Eagle Medium with 1% penicillin and streptomycin (control), (b) supplemented with 0.4 m*M* palmitate, (c) tumor necrosis factor α (TNFA), and (d) and 0.4 m*M* palmitate plus 20 ng/mL TNFA for 24 h. Cells were subsequently processed for live caspase 3/7 staining and immediately photomicrographed. Data are expressed as a percentage of hepatic cells that stained positively for caspase 3/7 (e). We also examined the potential alternation in size of nuclei (µm^2^) and found no significant (*P* > 0.05) difference among the groups (f). Data are expressed as the mean ± SEM of 2 independent experiments. Columns with different letters (A, B) differ significantly (*P* < 0.05; n = 4–8). +ve = positive.

**Figure 3:**
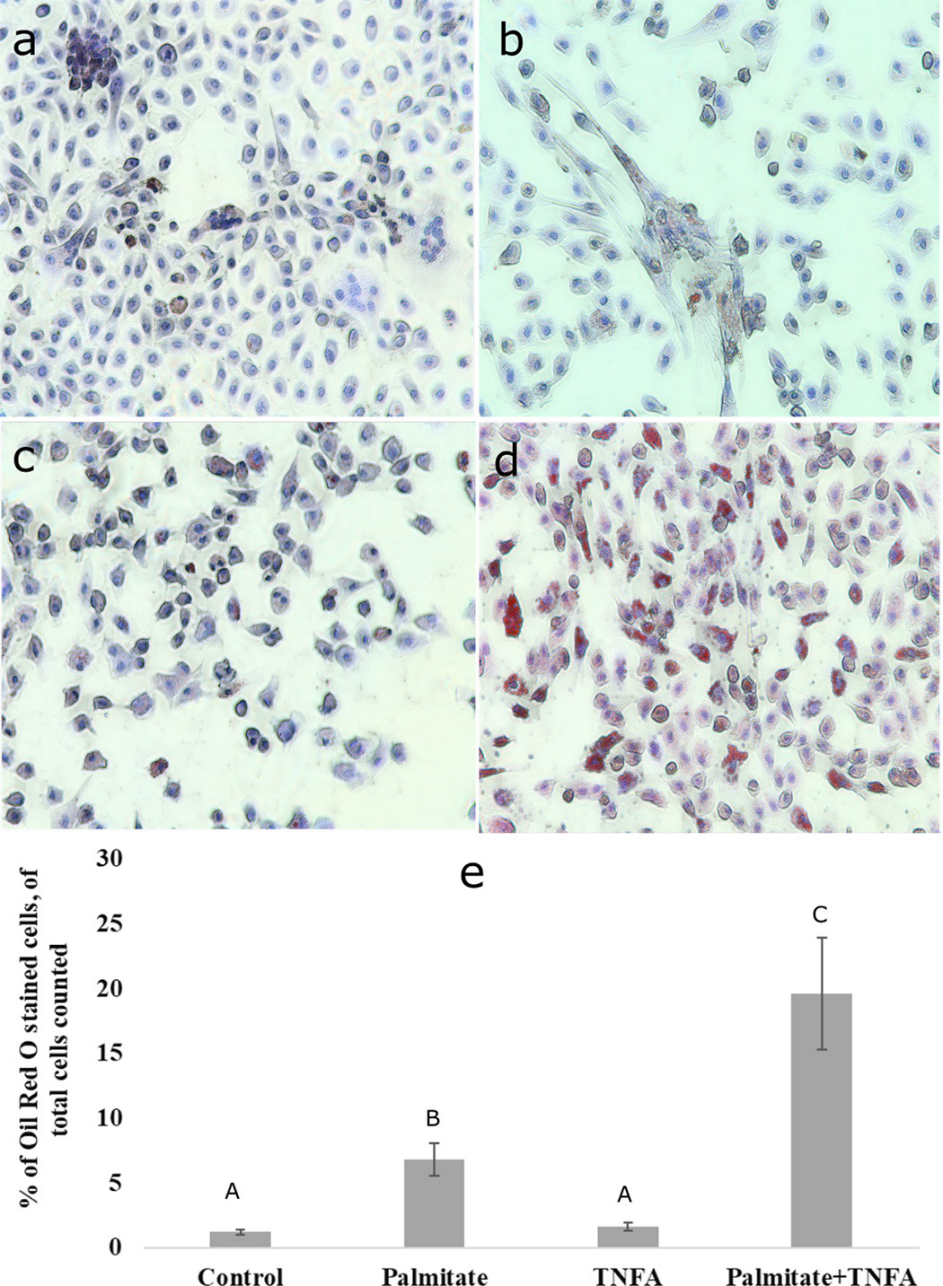
Lipid accumulation in bovine primary liver cells after 24 h of treatment using Oil Red O (EMD Millipore) staining. (a) Primary bovine hepatocytes were incubated in 10% fetal bovine serum/Dulbecco's Modified Eagle Medium with 1% penicillin and streptomycin (control), (b) cells supplemented with 0.4 m*M* palmitate, (c) tumor necrosis factor α (TNFA), and (d) 0.4 m*M* palmitate plus 20 ng/mL TNFA for 24 h. (e) Quantification of lipid-accumulating cells stained with Oil Red O. Data are expressed as a percentage (± SEM) of hepatic cells that stained positively for lipid accumulation. Columns with different letters (A–C) differ significantly (*P* < 0.05).

Moreover, another advantage of this in vitro model of bovine fatty liver occurs because the cell population is not purified to include only hepatocytes, but rather is a mixed cell culture that contains cells representative of the liver in vivo, allowing for physiological responses that should be more consistent than cultures that utilize purified hepatocytes alone. Moreover, our findings are consistent with bovine hepatic cell culture models exposed to NEFA previously used, furthering our confidence in our model (Li et al., 2020). Indeed, mixed cell cultures have been demonstrated to be superior to purified cell cultures in cases of disease modeling with primary cells (Buck et al., 2002; Serpero et al., 2009). Furthermore, this mixed cell culture model demonstrates robust proliferation of hepatocytes supporting long-term viability of the hepatocytes, which is lacking in previously described models.

In conclusion, we have developed a simple method for collecting readily available tissue from adult female animals rather than neonates (typically male) from the abattoir that should be more physiologically representative of in vivo hepatic lipidosis of the periparturient dairy cow than previously presented methods. This mixed cell culture method has the advantage of being more representative of physiological response, the simplicity of not requiring complex culture medium or perfusion techniques, and robust proliferation resulting in increased duration of cellular viability.
